# Philadelphia chromosome positive chronic myeloid leukemia with 5q deletion at diagnosis

**DOI:** 10.1186/s13039-021-00539-0

**Published:** 2021-03-08

**Authors:** Ahmed Maseh Haidary, Zeeshan Ansar Ahmed, Jamshid Abdul-Ghafar, Soma Rahmani, Sarah Noor, Farahnaz Erfani, Maryam Ahmad, Naeem Lakanwall, Haider Ali Malakzai, Abdul Sami Ibrahimkhil, Esmatullah Esmat, Mujtaba Haidari, Nimattullah Yousufzai, Samuel Sharif, Abdul Hadi Saqib

**Affiliations:** 1 Department of Pathology and Clinical Laboratory, French Medical Institute for Mothers and Children (FMIC), Kabul, Afghanistan; 2 Department of Pathology and Diagnostic Laboratory, Agha Khan University (AKU), Karachi, Afghanistan; 3 Department of Haemato-Oncology, Jumhoriat Hospital, Kabul, Afghanistan

**Keywords:** Philadelphia chromosome (Ph), Chronic myelogenous leukemia (CML), 5q deletion

## Abstract

**Background:**

Although, molecular genetic analyses became more and more important to guide therapy decisions in leukemia, banding cytogenetic analysis has retained its vital role in diagnosis and monitoring of chronic myeloid leukemia (CML), by quick and easy enabling identification of pathognomonic Philadelphia chromosome (Ph).

**Case presentation:**

A 45 year old female presented with characteristic hematological features of CML in chronic phase; cytogenetic studies revealed the presence of the typical Ph and a deletion of almost entire long arm of a chromosome 5.

**Conclusion:**

5q deletions have rarely been reported in CML. Those seen yet were either associated with tyrosine kinase inhibitor therapy or detected post allogeneic stem cell transplantation. To our knowledge, this is the first case of Ph positive CML accompanied by a 5q deletion.

## Introduction

Chronic myelogenous leukemia (CML), results from a balanced translocation t(9;22)(q34;q11.2) giving rise to the *BCR-ABL1* chimeric gene being the oncogenic driver of CML [1]. This fusion gene is not only pathognomonic diagnostic marker of but also therapeutic target for CML [2]. Since the introduction of Imatinib as the first medication with tyrosine kinase inhibitor (TKI) activity there was continuous advancement in CML management, not only by refinement of diagnostic and monitoring modalities but also by introduction of multiple generations of TKI agents, leading to better disease outcomes [3, 4]. Besides cytogenetics, molecular cytogenetics is an essential pillar for the diagnosis and monitoring of patients with CML [5]. Quantitative polymerase chain reaction (Q-PCR) to detect low levels of *BCR-ABL1* fusion gene presence has now enabled for deeper scrutiny into the disease, allowing to identify abnormal clones as small as 1 in 10,000 cells [6, 7]. Accordingly, patients achieving and maintaining deep molecular response (i.e. having a negative Q-PCR-test result) are entitled for complete discontinuation of TKI therapy [8]. Although, the mentioned molecular achievements in diagnosis and monitoring of CML have been essential for progress in disease management, the role of cytogenetic studies is undeniably still significant, as those allow for identification of additional chromosome abnormalities of prognostic significance [9]. This is as additional cytogenetic abnormalities may be acquired during course of disease and/or therapy, i.e. clonal evolution may take place [10, 11]. Such additional cytogenetic abnormalities may have significant effect on the disease profile and response to therapy [12].One of the rarely seen additional cytogenetic abnormalities is deletion in the long arm of a chromosome 5 (5q-); however, this has been reported yet only in CML-patients under during therapy [13, 14].

Here we present a case with classic clinical and hematological features of CML in chronic phase where banding cytogenetics revealed presence of a Philadelphia chromosome (Ph) along with deletion del(5)(q13.3).

## Case presentation

**Fig. 1:**
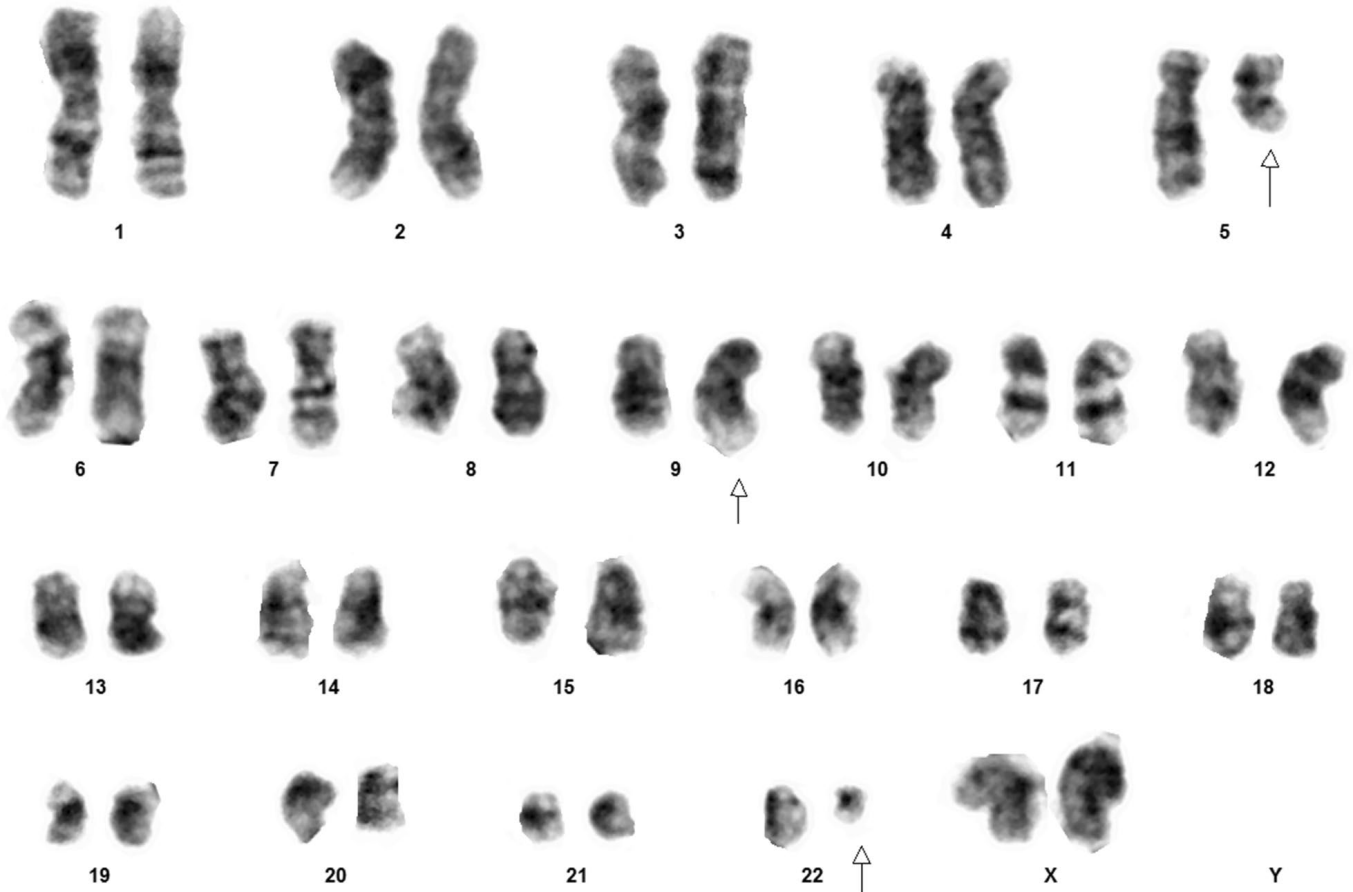
A typical metaphases of the reported patient showing a karyotype of 46,XX,del(5)(q13.3),t(9;22)(q34;q11.2)

A 45-year old female, without any significant past medical illness, presented with lethargy, anorexia and pallor, which progressively developed over 6 months. On examination, the patient was mildly pale, and abdominal examination revealed moderately enlarged spleen without hepatomegaly. Complete blood count (CBC) revealed moderate anemia, hyperleukocytosis (130 × 10^3^ white blood cells per microliter), demonstrating predominance of granulopoesis with bimodal peak of mature neutrophils (51%) and myelocytes (29%). The blast count was less than 1% and there was no basophilia. With an initial suspected diagnosis of CML cytogenetic analysis was performed. Peripheral blood was sampled for conventional non-phytohemagglutinine-stimulated karyotyping. All 20 analyzed metaphases revealed a karyotype 46,XX,del(5)(q13.3),t(9;22)(q34;q11.2) (Fig. 1). After confirming suspicion of CML by this, TKI therapy was initiated, under which the patient does well up to now.

## Discussion

CML, once an indefinitely progressive hematological malignancy, is now a success story in the field of hemato-oncology [15]. Patients, when treated properly, now achieve complete molecular remission within weeks to months, thanks to the advent of TKI medication [1, 16–18]. All current guidelines aim for close patient monitoring with quantitation of disease burden, to identify patients for whom TKI therapy needs to be upgraded to more potent TKI agents [9, 19, 20]. Moreover, mutational studies allow for identification of patients for whom aggressive protocols need to be considered right from the beginning [21].

However, still banding cytogenetic analysis plays an important role in first diagnostics and management of CML [22]. Besides detection of Ph chromosome, complex chromosomal abnormalities can be identified, which may determine the patient as candidate for alternative and more aggressive therapeutic options, sometimes even leading to allogeneic stem cell transplantation [23–25].

Acquisition of additional cytogenetic abnormalities, including 5q-, can be one of the markers for clonal evolution, thus warranting patient re-evaluation that could possibly change the disease management plan [11, 26]. While chromosome 5q deletion has good prognostic implication in patients with de-novo myelodysplastic syndrome (MDS), when identified in patients with therapy related or de-novo acute myeloid leukemia, the prognosis is poor [27, 28]. 5q deletion in CML has been reported only rarely in patients yet during therapy with conventional TKI or after stem cell transplantation [13, 14]. Similarly, there have been cases that were initially diagnosed and managed as MDS associated with 5q deletion, who ultimately transformed to Ph + CML while retaining the original 5q deletion in the novel malignant clones [13]. Our patient, who presented with characteristic clinical and hematological features of CML in chronic phase, was initially a healthy individual, suggesting rather that the 5q- was acquired most likely after Ph chromosome appeared in the bone marrow cell clone.

## Conclusion

To our knowledge our patient was the first case of Ph + CML with 5q deletion at diagnosis. Therapeutic and prognostic implication of such a presentation would require further evaluation, including close follow-up.

## Data Availability

All generated data is included in this article.

## Abbreviations

ABL1: Abelson gene 1
BCR: Break point cluster region
CBC: Complete blood count
CML: Chronic myeloid leukemia
MDS: Myelodysplastic syndrome
Ph: Philadelphia chromosome
Q-PCR: Quantitative polymerase chain reaction
TKI: Tyrosine kinase inhibition

## References

[1] Haidary AM, Azma RZ, Ithnin A, Alauddin H, Tumian NR, Tamil AM, Razak NFA, Abu Amis SH, Zin NM, Shuib S (2019). FISH versus real-time quantitative PCR for monitoring of minimal residual disease in chronic myeloid leukaemia patients on tyrosine kinase inhibitor therapy. Malays J Pathol.

[2] Van Etten RA (2004). Mechanisms of transformation by the BCR-ABL oncogene: new perspectives in the post-imatinib era. Leuk Res.

[3] Branford S, Rudzki Z, Harper A, Grigg A, Taylor K, Durrant S, Arthur C, Browett P, Schwarer AP, Ma D, Seymour JF, Bradstock K, Joske D, Lynch K, Gathmann I, Hughes TP (2003). Imatinib produces significantly superior molecular responses compared to interferon alfa plus cytarabine in patients with newly diagnosed chronic myeloid leukemia in chronic phase. Leukemia.

[4] Loren CP, Aslan JE, Rigg RA, Nowak MS, Healy LD, Gruber A, Druker BJ, McCarty OJ (2015). The BCR-ABL inhibitor ponatinib inhibits platelet immunoreceptor tyrosine-based activation motif (ITAM) signaling, platelet activation and aggregate formation under shear. Thromb Res.

[5] Hehlmann R, Saußele S, Voskanyan A, Silver RT (2016). Management of CML-blast crisis. Best Pract Res Clin Haematol.

[6] Cross NC, White HE, Müller MC, Saglio G, Hochhaus A (2012). Standardized definitions of molecular response in chronic myeloid leukemia. Leukemia.

[7] Hughes TP, Ross DM (2016). Moving treatment-free remission into mainstream clinical practice in CML. Blood.

[8] Kumagai T, Nakaseko C, Nishiwaki K, Yoshida C, Ohashi K, Takezako N, Takano H, Kouzai Y, Murase T, Matsue K, Morita S, Sakamoto J, Wakita H, Sakamaki H, Inokuchi K, Kanto CML, Shimousa Hematology Study Groups (2018). Dasatinib cessation after deep molecular response exceeding 2 years and natural killer cell transition during dasatinib consolidation. Cancer Sci..

[9] Swerdlow SH, Campo E, Harris NL, Jaffe ES, Pileri SA, Stein H, Thiele J, Arber DA, Hasserjian RP, Le Beau MM, Orazi ASR (2017). World health organization classification of tumours of haematopoietic and lymphoid tissues.

[10] Zámecníkova A, Krizana P, Gyarfás J, Vahancík A (2001). Philadelphia-positive chronic myelogenous leukemia with a 5q- abnormality in a patient following interferon-alpha therapy. Cancer Genet Cytogenet.

[11] Wang W, Cortes JE, Tang G, Khoury JD, Wang S, Bueso-Ramos CE, DiGiuseppe JA, Chen Z, Kantarjian HM, Medeiros LJ, Hu S (2016). Risk stratification of chromosomal abnormalities in chronic myelogenous leukemia in the era of tyrosine kinase inhibitor therapy. Blood.

[12] Krishna Chandran R, Geetha N, Sakthivel KM, Suresh Kumar R, Jagathnath Krishna KMN, Sreedharan H (2019). Impact of additional chromosomal aberrations on the disease progression of chronic myelogenous leukemia. Front Oncol.

[13] Zaslav A-L, Gupta R, Burks BT, Schuster M, Jalilizeinali B, Knorr E, Tully D, Fernicola P, Mercado T, Spitzer SG, Golighty MG, Ma Y, Ahmed T (2016). Transformation of myelodysplastic syndrome with isolated 5q-syndrome to chronic myelogenous leukemia with a novel complex BCR/ABL1 translocation with rapid progression to blast crisis. Hematol Leuk.

[14] Lambertenghi Deliliers G, Annaloro C, Pozzoli E, Oriani A, Della Volpe A, Soligo D, Lambertenghi Deliliers D, Tagliaferri E, Bertolli V, Romitti L (1999). Cytogenetic and myelodysplastic alterations after autologous hemopoietic stem cell transplantation. Leuk Res.

[15] Soligo D, Romitti L, Bertolli V, Della Volpe A, Annaloro C, Lambertenghi DG (1995). 5q- in a case of chronic myelogenous leukemia relapsed after allogeneic bone marrow transplantation. Haematologica.

[16] Yeung CC, Egan D, Radich JP (2016). Molecular monitoring of chronic myeloid leukemia: present and future. Expert Rev Mol Diagn.

[17] Mensink E, van de Locht A, Schattenberg A, Linders E, Schaap N, Geurts van Kessel A, De Witte T (1998). Quantitation of minimal residual disease in Philadelphia chromosome positive chronic myeloid leukaemia patients using real-time quantitative RT-PCR. Br J Haematol.

[18] Jabbour E, Kantarjian H (2016). Chronic myeloid leukemia: 2016 update on diagnosis, therapy, and monitoring. Am J Hematol.

[19] Soverini S, De Benedittis C, Mancini M, Martinelli G (2016). Present and future of molecular monitoring in chronic myeloid leukaemia. Br J Haematol.

[20] Usui N. Updated European leukemianet recommendations for the management of CML. In: Molecular pathogenesis and treatment of chronic myelogenous leukemia; 2015. pp. 81–100.

[21] Shamroe CL, Comeau JM (2013). Ponatinib: A new tyrosine kinase inhibitor for the treatment of chronic myeloid leukemia and Philadelphia chromosome-positive acute lymphoblastic leukemia. Ann Pharmacother.

[22] Jabbour E, Kantarjian H (2018). Chronic myeloid leukemia: 2018 update on diagnosis, therapy and monitoring. Am J Hematol.

[23] Fabarius A, Kalmanti L, Dietz CT, Lauseker M, Rinaldetti S, Haferlach C, Göhring G, Schlegelberger B, Jotterand M, Hanfstein B, Seifarth W, Hänel M, Köhne CH, Lindemann HW, Berdel WE, Staib P, Müller MC, Proetel U, Balleisen L, Goebeler ME, Dengler J, Falge C, Kanz L, Burchert A, Kneba M, Stegelmann F, Pfreundschuh M, Waller CF, Spiekermann K, Brümmendorf TH, Edinger M, Hofmann WK, Pfirrmann M, Hasford J, Krause S, Hochhaus A, Saußele S, Hehlmann R, SAKK and the German CML Study Group (2015). Impact of unbalanced minor route versus major route karyotypes at diagnosis on prognosis of CML. Ann Hematol.

[24] Kim YJ, Kim DW, Lee S, Kim HJ, Kim YL, Hwang JY, Oh IH, Park YH, Lee YK, Min CK, Kim TG, Han TH, Min WS, Kim CC (2002). Comprehensive comparison of FISH, RT-PCR, and RQ-PCR for monitoring the BCR-ABL gene after hematopoietic stem cell transplantation in CML. Eur J Haematol.

[25] Malakzai HA, Rahmani S, Haidary AM, Noor S, Ahmad M, Ibrahimkhil AS, Sharif S (2020). Complex cytogenetic abnormalities in chronic myeloid leukemia resulting in early progression to blast crisis: a case report. J Med Case Rep.

[26] Horne SD, Stevens JB, Abdallah BY, Liu G, Bremer SW, Ye CJ, Heng HH (2013). Why imatinib remains an exception of cancer research. J Cell Physiol.

[27] Steensma DP (2018). Myelodysplastic syndromes current treatment algorithm 2018. Blood Cancer J.

[28] Vardiman J, Reichard K (2015). Acute myeloid leukemia with myelodysplasia-related changes. Am J Clin Pathol.

